# Evaluation of Physiological Coping Strategies and Quality Substances in Purple SweetPotato under Different Salinity Levels

**DOI:** 10.3390/genes13081350

**Published:** 2022-07-27

**Authors:** Xin Wang, Wei-Wei Dai, Chong Liu, Guang-Xi Zhang, Wei-Han Song, Chen Li, Yuenden-Ci Yangchen, Run-Fei Gao, Yu-Yu Chen, Hui Yan, Wei Tang, Meng Kou, Yun-Gang Zhang, Bo Yuan, Qiang Li

**Affiliations:** 1 Sweetpotato Research Institute, Chinese Academy of Agricultural Sciences/Xuzhou Institute of Agricultural Sciences in Jiangsu Xuhuai District, Key Laboratory of Biology & Genetic Breeding of Sweetpotato, Ministry of Agriculture and Rural Affairs, Xuzhou 221131, China; xznkywx@163.com (X.W.); xzsongweihan@126.com (W.-H.S.); 1020180010@jsnu.edu.cn (C.L.); 15852141027@163.com (R.-F.G.); chen1262066842@163.com (Y.-Y.C.); yanhui_sweetpotato@163.com (H.Y.); tangweilhr@163.com (W.T.); koumeng2113@163.com (M.K.); zyga1@126.com (Y.-G.Z.); 2 School of Life Science, Jiangsu Normal University, Xuzhou 221116, China; daiww1998@163.com (W.-W.D.); zhang2019728@163.com (G.-X.Z.); yj13897790251@163.com (Y.-C.Y.); 3 Institute of Agricultural Sciences in the Coastal Area Jiangsu, Yancheng 224002, China; cellbio@163.com

**Keywords:** salinity, purple sweetpotato, quality analysis, metabolites, gene

## Abstract

Although salinity stress is one of the principal abiotic stresses affecting crop yield, a suitable concentration of NaCl has proven to be useful for increasing crop quality. This study used low salinity (34 mmol/L NaCl) and high salinity (85 mmol/L) to cultivate purple sweetpotato. Using transcriptomics and metabolomics to profile the pathway indicated that glycometabolism, secondary metabolite biosynthesis and the starch catabolic process were the significant pathways under the salinity stress. Further research showed that purple sweetpotato could regulate genes related to the regulation of the cellular Na^+^, K^+^, and other ions concentration in response to the low salinity tolerance, but loses this ability under high salinity. Meanwhile, under low salinity, the activity of antioxidant enzymes and their related gene expression are maintained at a high level. The low salinity influences the monosaccharide composition as well as the content and regulation of genes related to starch synthesis. Quality analysis showed that the low salinity could increase the starch content and influence the amylopectin biosynthesis. It suggested that low salinity promotes substance accumulation. High salinity could increase the anthocyanins biosynthesis and low salinity had a significant impact on phenolic acid and flavonol. Finally, the gene expression levels also prove the low salinity could change the composition and content level of the purple sweetpotato. This study showed that an appropriate concentration of NaCl can be used as an elicitor for application in purple sweetpotato planting.

## 1. Introduction

Salts can exist in soil naturally or by various adding processes (irrigation or fertilizers). As we know, soil salinity is a major environmental challenge. Saline soil accounts for approximately 7% of the area in the world, and the phenomenon is becoming more and more serious [1,2]. It causes severe crop revenue loss all over the world. Salt-induced osmotic, ionic, and oxidative stress are responsible for plant suffering. Soil salinity adversely affects the physiology, morphology, and biochemical processes of plants, including the uptake of water and nutrients and the germination of seeds. Properly speaking, soil salinity could cause osmotic stress, oxidative stress, nutrient deficiency (macro and microelements), and ion toxicity [3,4,5,6].

Meanwhile, salinity stress affects the photosynthesis, signal transduction, protein synthesis, and degradation processes in plants and inhibits crop yield dramatically. Some studies showed that high salinity could reduce the leaves’ net photosynthetic rate and carbohydrate metabolism-related enzymes’ activities and lead to the inhibition of carbon assimilation [3,4,5,6]. In addition, salinity stress also drastically affects nitrogen metabolism, carbon metabolism, and plant nutrition [7]. However, plants have developed multiple and complex strategies to adapt to salinity stress at the molecular, cellular, physiological, and biochemical levels [8,9,10,11]. The prime example is the synthesis and accumulation of low-molecular-weight organic compounds in the cytosol and organelles [12]. Plants can produce and accumulate compounds called compatible osmolytes, including simple sugars, disaccharides, sugar alcohols or polyols, amino acids, and plutonium compounds [13,14]. Meanwhile, numerous genes involved in many signaling pathways are induced in response to stresses, including functional and regulatory genes. These genes increase plant stress tolerance and control gene expression via signaling pathways. Those functional genes mainly encode relevant reactive enzymes for the biosynthesis pathway, such as proline biosynthesis, scavengers of antioxidants or reactive oxygen species (ROS), and molecular chaperones. The regulatory genes that play important roles in signal transduction and gene expression include calcium sensors, membrane-localized receptors and protein kinases [15].

Omics technology, including metabolomics and transcriptome, has become increasingly crucial in systemically exploring the mechanisms of plant growth and development and the response to biotic and abiotic stresses [1,16]. For example, metabolomics revealed that the content of aspartate clearly increased and that γ-aminobutyric acid levels decreased gradually. In contrast, organic acid did not show an obvious change under salt stress [17]. The correlation analysis of omics showed more information on how the plant adapts the abiotic stresses. Comparing the transcriptome and metabonomics profiles in response to abiotic stresses is applicable to understanding which responses are specific to a given stress, or represent shared responses to the various stresses [18].

Sweetpotato (*Ipomoea batatas* (L.) Lam.), a dicotyledonous plant of the family *Convolvulaceae*, is the staple event crop worldwide. Sweetpotato storage roots and other aerial parts of the plant are used as human food, animal feed, and industrial products [19,20]. As one of the varieties, Purple sweetpotato has been widely planted in China and other Asian countries. Purple sweetpotato is popular among consumers as a functional food because of its high contents of anthocyanins and other polyphenols. Some studies showed that a series of physiological and biochemical catabolisms, including nutrition content, pigmentation, and unique flavor were changed under salt stress. These studies also showed that salt stress influences sweetpotato growth, fresh weight, health-promoting compounds and antioxidant activity. In a previous study, we found that low salinity stress could increase the saccharinity of sweetpotato. These changes also reflected the quality of the sweetpotato. However, the related metabolism and gene expression has not been thoroughly studied. The present study has been conducted to compare the metabolism and gene expression in purple sweetpotato to understand the quality control response to both low and high salinity.

## 2. Materials and Methods

### 2.1. Plant Materials

The purple sweetpotato cultivar Xuzishu-8 was selected from Yancheng, China as the experimental material. The storage root was obtained from two saline-alkali soils with different NaCl concentrations, including 2‰ (low salinity) and 5‰ (high salinity). The fresh storage root was chosen and dried at 70 °C for 24 h to calculate dry weight.

### 2.2. Transcriptome Analysis

Total RNA was extracted from the storage root samples ground in liquid nitrogen using a modified phenol/chloroform/isoamyl alcohol (P:C:I = 25:24:1, *v*/*v*) mediated extraction protocol [21].

### 2.3. Metabolite Extraction and Profiling Analysis

We collected 500 mg samples of the storage root and placed them in 1.5 mL Eppendorf tubes, mixed them with 0.5 mL of methanol as an extraction liquid, and added 50 μL of 2-chlorophenyl alanine (1 mg/mL stock in dH_2_O) as an internal standard. The sample was then vortex-mixed for 30 s, stored at −20 °C for 60 min, and centrifuged for 15 min at 12,000 rpm at 4 °C. The supernatant was transferred into a fresh 2 mL LC–MS glass vial and analyzed using Quadrupole Time-of-flight Mass Spectrometry (Q-TOF MS). The chromatographic mobile phase was an aqueous solution containing 0.1% formic acid (A) and acetonitrile (B). The gradient elution procedure for determination of samples was as follows: 0–5 min, 95% A; 5–10 min, 95–50% A; 10–15 min, 50–0% A; 15–20 min, 0%A, 5 μL per injection, flow rate 0.3 mL/min. Chromatographic column: Phenomenex Kinetex C18, 2.1 × 30 mm, chromatographic column temperature: 30 °C. Data were collected in electrospray ionization (ESI) positive and negative ion modes, spray voltage: 3000 V; evaporation temperature: 450 °C; scanning range: 70–1050 *m*/*z*.

The identification of endogenous metabolites and data processing were performed as described in a previous study [22]. MetaboAnalyst 4.0 software (http://www.metaboanalyst.ca/, USA) was used to analyze the difference between groups, and to identify the endogenous metabolites with variable importance in projection (VIP) values greater than 1, and to plot the Principal Component Analysis (PCA), Partial Least Squares Discriminant (PLS-DA), and topological analysis charts.

### 2.4. K^+^, Na^+^ Concentration Determination

Dried purple sweetpotato samples were ground and mixed, then digested with H_2_SO_4_-H_2_O_2_, LSCB K^+^ and Na^+^ were measured using an atomic absorption spectrophotometer according to the method described in Yang [23].

### 2.5. Related Enzymes Activity Assay

Enzymes were extracted from 0.5 g fresh sample of the storage root using a mortar and pestle with 5 mL extraction buffer containing 50 mM potassium phosphate buffer (pH 7.6) and 0.1 mM Na-EDTA. The homogenate was centrifuged at 15,000× *g* for 15 min and the supernatant fraction was used to assay the various enzymes. All steps in the preparation of enzyme extracts were performed at 4 °C. Then, the SOD, CAT, CAT and GPX activities were assayed according to Kit instructions (Biyuntian, China).

The extraction of enzymes from purple sweetpotato was performed in triplicate, and the enzyme activity of each extract was measured once, taking into account a specific blank sample. The method to quantify α and β-amylase was adapted to allow us to distinguish between free and total α/β-amylase activity by the addition of 100 mM cysteine to the extraction buffer. The potential activity of the bound α/β-amylase was calculated as the difference between total α/β-amylase activity (presence of 100 mM cysteine) and free α/β-amylase activity (no cysteine present).

### 2.6. Measurement of Soluble Sugar, Monosaccharide, and Starch Content

The carbohydrates in the storage root were extracted from dry samples of the storage root (0.1 g) using 10 mL 80% (*v*/*v*) ethanol. The residues were extracted with 20 mL 2 mol/L perchloric acid, the volumes were adjusted to 50 mL in volumetric flasks, and the supernatants were analyzed to determine the starch contents by anthrone colorimetry. Separation of amylose and amylopectin using the method in our lab and determined the contents through standard cure equation, *y =* 0.018*x* − 0.017, R^2^ = 0.9945 and *y =* 0.074*x* − 0.029, R^2^ = 0.9983, respectively.

The supernatants were analyzed for fructose, maltose, saccharose, glucose, xylose, and rhamnose contents by High-Performance Liquid Chromatography (HPLC) coupled with an evaporative light scattering detector (ELSD). The chromatographic condition is as follows: the mobile phase was water with a flow rate of 1.0 mL/min, and the chromatographic column was Bio-Rad Aminex. Standard monosaccharides were used to calculate the content through the equation of standard cure. Fructose: *y* = 10.572*x* − 874.12, R^2^ = 0.9992. xylose: *y =* 9.762*x* − 543.95, R^2^ = 0.9997. Maltose: *y =* 28.77*x* − 445.92. R^2^ = 0.9987. Saccharose: *y =* 11.74*x* − 281.00, R^2^ = 0.9969. Glucose: *y =* 75.95*x* − 331.63, R^2^ = 0.9990. Xylose: *y =* 43.84*x* − 325.77, R^2^ = 0.9984. Rhamnose: *y =* 34.54*x* − 417.39, R^2^ = 0.9985. The soluble sugars in the storage root were extracted using fresh samples (about 0.5 g) that were boiled for 1 h in 25 mL water, and the supernatants were analyzed by anthrone colorimetry.

We used GC–MS to the determination of other monosaccharides, including D-fructose, inositol, D-galactose, trehalose, xylitol, fucose, arabinose, and sorbitol. To conduct standard curve, all standards solutions were diluted with Acetonitrile (ACN) to 0.5 ppm. The powders freeze-dried storage root were diluted to a mixed liquid of methanol, isopropanol, and water. The extracts were centrifuged and supernatants were collected. The extracts were added to the internal standard, evaporated by a nitrogen gas stream, and transferred to the lyophilizer for freeze-drying. For derivatization, the residue was mixed with a solution of methoxyamine hydrochloride in pyridine. After that, BSTFA was added to the mixture, vortex-mixing. Finally, n-hexane diluted the mix for GC–MS/MS analysis. The GC–MS/MS method was according as the following. GC–MS/MS analysis: Agilent 7890B gas chromatography coupled to a 7000D mass spectrometer and a DB-5MS column (30 m length × 0.25 mm i.d. × 0.25 μm film thickness, J&W Scientific, Folsom, CA, USA) was used to GC–MS/MS analysis of sugars. Helium was used as carrier gas at a flow rate of 1 mL/min. The injection volume was 3 μL and the front Inlet mode was 3:1. The oven temperature was held at 170 °C for 1 min, and then raised to 250 °C at 10 °C/min, raised to 280 °C at 4 °C/min, raised to 310 °C at 25 °C/min and held at the temperature for 3.72 min. All samples were analyzed in both full scan (mass range of 40–510 amu) and selective ion scan mode. The injector inlet and transfer line temperatures were 250 and 240 °C, respectively.

### 2.7. Determination of the Composition and Content of Polyphenols in Storage Root

A total of 100 mg of freeze-dried storage root powder was extracted with 1.0 mL of 70% methanol at 4 °C overnight. After centrifugation at 10,000 g for 10 min, the supernatant was analyzed using a liquid chromatography electrospray ionization tandem mass spectrometry (LC−ESI−MS/MS) system (HPLC, Shim-pack UFLC SHIMADZU CBM30A system; MS, Applied Biosystems 4500 Q TRAP) with a Waters ACQUITY UPLC HSS T3 C18 column (1.8 μm, 2.1 × 100 mm). Solvents A (0.04% acetic acid in water, *v*/*v*) and B (0.04% acetic acid in acetonitrile) were used with the following gradient: 95% A gradient descent to 0% in 15 min and keep proportion for 5 min. The flow rate was 0.4 mL/min and the column was 40 °C. The effluent was connected to an ESI-triple quadrupole-linear ion trap (Q TRAP)−MS. Linear ion trap (LIT) and triple quadrupole (QQQ) scans were acquired on a triple quadrupole-linear ion trap mass spectrometer (Q TRAP), API 4500 Q TRAP LC/MS/MS system, equipped with an ESI turbo ion spray interface, operating in the positive-ion mode, and controlled by Analyst 1.6.3 software (AB Sciex, Redwood City, CA, USA). The ESI source operation parameters were as follows: ion source, turbo spray; source temperature, 550 °C; ion spray voltage, 5500 V; ion source gas I, 55 psi; ion source gas II, 55 psi; curtain gas, 25.0 psi; and collision gas, 12 psi. Instrument tuning and mass calibration were performed with 10 and 100 μmol L−1 polypropylene glycol solutions in QQQ and LIT modes, respectively. QQQ scans were acquired as multiple reaction monitoring (MRM) experiments with a collision gas (nitrogen) set to 5 psi. DP and CE for individual MRM transitions were performed with further DP and CE optimization. A specific set of MRM transitions were monitored for each period according to the metabolites eluted within this period.

### 2.8. Validation Experiment

Determination of related gene as following method: For quantitative real-time polymerase chain reaction (RT-PCR) analysis, total RNA was extracted using an RNAiso Plus kit (Takara, Beijing, China). A TURBO DNA-free kit (Sigma–Aldrich, Shanghai, China) was used to remove genomic DNA. Single-stranded cDNA was synthesized using a PrimeScriptH RT kit (Takara). The SYBR green method (Takara) was performed on a CFX96 Touch real-time PCR detection system. The relative expression levels of the amplified products were calculated using the 2^−ΔΔCt^ method. Primers for the selected unigenes were designed online using NCBI Primer-BLAST (Bio-Rad, Hercules, CA, USA). CsActin (HQ420251.1) was used as the internal housekeeping gene (Table 1). The primer design was according to the previous study [24,25].

### 2.9. Statistical Analysis

Data were obtained from three independent biological replicates. Statistical analysis was conducted using Minitab 17.0 statistical software (Minitab, Inc., Coventry, UK). One-way analysis of variance (ANOVA) with grouping information and Fisher’s least significant difference (LSD) test were used at the 5% level.

## 3. Results

### 3.1. Transcriptome Analysis

**Figure 1:**
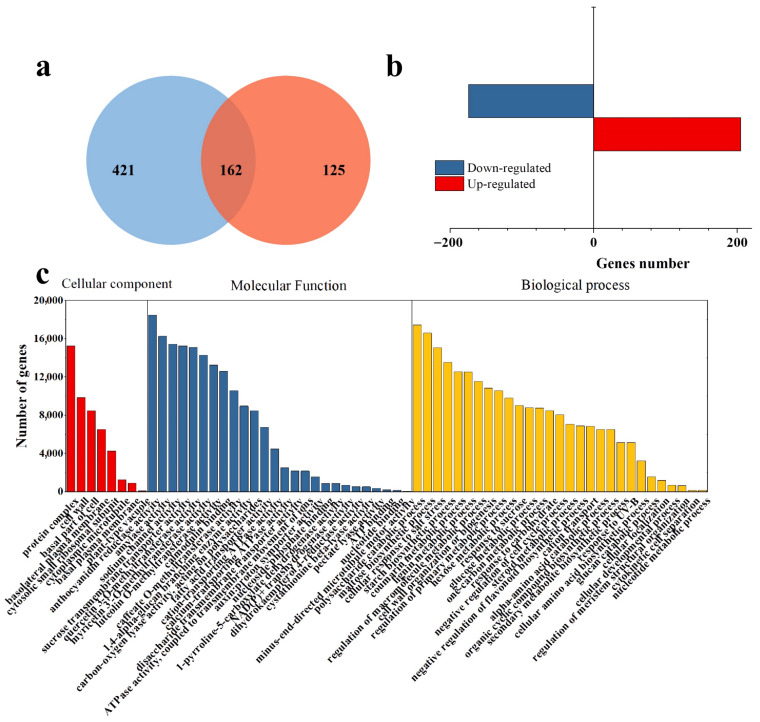
The transcriptome analysis result. (**a**) Venn diagram of DEGs; (**b**) The up- and down-regulated gene numbers in DEGs; (**c**) GO functional classification of DEGs between low salinity and high salinity.

The analysis of transcriptomic profiling in response to different soil salinity levels. A total of 546 differentially expressed sequences were obtained using the DEGseq under different saline soil conditions (Figure 1). Out of these, a total of 379 (including 205 up-regulated DEGs and 174 down-regulated DEGs) were successfully annotated. The GO annotations showed that all enriched differentially expressed genes were classified into three categories: biological process, cellular components, and molecular function. Most differentially expressed genes were involved in the biological process in response to the biosynthesis of carbohydrate and secondary metabolites, including flavone, lignin, hexose, starch, and sucrose metabolic processes. Most specific differentially expressed genes were annotated in the molecular function category to the GO terms of protein activity on the sugar and secondary biosynthesis process. Most differentially expressed genes were assigned to the protein complex and cell wall for the cellular component category. The most pronounced differentially expressed genes were annotated to anthocyanidin and other flavone biosynthesis processes.

**Figure 2:**
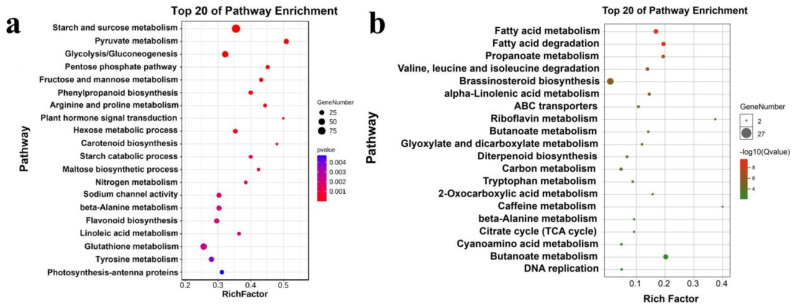
Analysis of KEGG Enrichment of differentially expressed genes in purple sweetpotato under salinity. (**a**) The top 20 of pathway enrichment based on biological process and (**b**) based on molecular function.

The KEGG database screened the main pathway involved in different genes and showed the result in Figure 2. As shown in Figure 2a, the up-regulated genes mainly focused on starch and sucrose metabolism, pyruvate metabolism, and glycolysis/gluconeogenesis. Meanwhile, β-alanine metabolism, flavonoid biosynthesis and glutathione metabolism were significant pathways in the up-regulation. Brassinosteroid biosynthesis, fatty acid metabolism, and degradation were the main pathways involved in down-regulated genes.

The salinity also significantly influences the secondary metabolite biosynthesis and metabolism through the enrichment pathway and metabolism, such as anthocyanidin reductase activity (Figure 1c), flavonoid biosynthesis, and phenylpropanoid biosynthesis (Figure 2a). As we know, anthocyanidin, the primary and essential metabolite in the purple sweetpotato, plays a crucial role in antioxidant or other bioactivities. Flavonoid biosynthesis and phenylpropanoid biosynthesis were the primary metabolic pathways for anthocyanidin and other flavonoids [26]. It might be suggested that salinity could influence anthocyanidin biosynthesis. Meanwhile, as a starch-producing plant, starch, and other carbohydrates were the primary focus metabolites. In this study, salinity is the main influence on the starch biosynthetic process (Figure 1c). The enrichment pathway result points out that salinity positively regulates starch and sucrose metabolism and the catabolic process (Figure 2a). This means that the salinity might promote starch biosynthesis or metabolism.

### 3.2. The Effect of Salinity on the Metabolic Profile Variations

This section provides details on the analysis and comparison of variations in metabolites using LC–QTOFMS. A total of 171 metabolites, classified into 17 categories based on KEEG, were identified. The samples were used in the principal component analysis (PCA) model. The QC samples were locked in the center and tightly clustered, indicating optimum data quality and experimental stability. All samples in different groups were significantly separated, suggesting that the purple sweetpotato responds differently to different salt concentrations to generate different metabolites.

**Figure 3:**
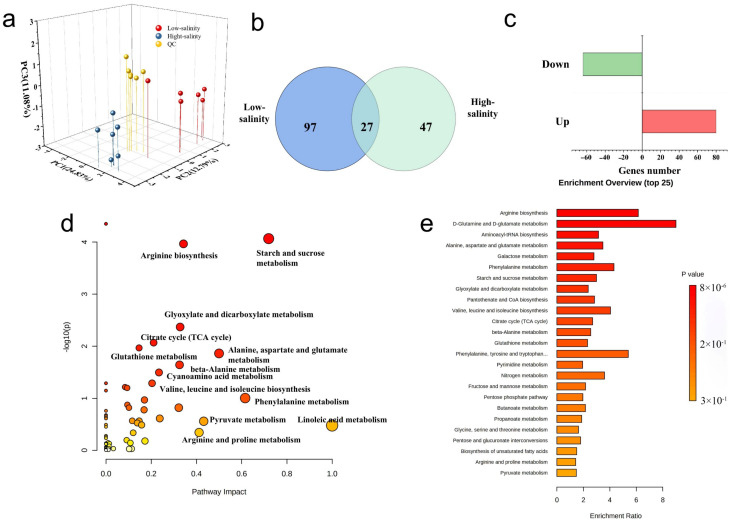
Differential metabolic profiles in purple sweetpotato in low and high salinity groups. (**a**) 3D-PLS-DA score plot; (**b**) Venn diagram of differential metabolites; (**c**) The up- and down-regulated gene numbers in differential metabolites; (**d**) The topological analysis of related metabolic pathways with MetaboAnalyst 4.0; (**e**) The enrichment analysis of the comparison between low and high salinity. All data are representative of at least three independent experiments. Statistical significance was determined using a one-way ANOVA followed by Tukey’s post-hoc test.

Based on the analysis, 118 differentially metabolites (41 up and 87 down) by compared the differential salinity treatment (Figure 3b,c). Pathway and enrichment analyses were carried out to ascertain the effects of different salinity levels on the metabolic pathways of purple sweetpotato. As shown in Figure 3d,e, when purple sweetpotato was treated with low salinity, the perturbed metabolites were mainly related to carbohydrate metabolism, including starch and sucrose metabolism, and glyoxylate dicarboxylate metabolism. The enrichment of pathways also reflects the similar result of the metabolic pathway. Meanwhile, amino acid metabolisms were also the significant pathway involved under salinity. We chose 31 of them quantitatively by LC–MS/MS and list the fold change in the Table 2. More amino acid levels in purple sweetpotato were elevated in response to salinity. They included, proline (4.06, 8.72), glycine (6.91, 10.05), valine (29.8, 40.7), alanine (12.60, 18.05), aspartic acid (2.64, 4.72), serine (5.93, 18.05), cysteine (6.91, 11.38), phenylalanine (4.06, 7.00), lysine (7.08, 8.72), tryptophan (12.60, 15.39) and cysteine (15.44, 18.05). However, some other amino acid levels were reduced under low salinity. These included 3-aminoisobutty acid, aminomalonic acid, β-alanine, 3-hydroxypropionic acid, ornithine and tyrosine. The metabolites change was verified by the result of the transcriptomics above and point out that low salinity might influence the sugar metabolism. For example, as shown in the influenced metabolic pathway, the starch and sucrose metabolism were significantly affected by the salinity. This result was also following the previous transcriptome results. Meanwhile, the glyoxylate and dicarboxylate metabolism and glutathione metabolism refer to exogenous oxidative stress, such as salinity. It reveals that the low salinity might be influenced by the oxidative stress process or the synthesis of related antioxidants in response to salinity stress. This result also corresponded with the transcriptome result. It clearly demonstrates that the salinity can significantly influence ion transport, oxidative stress carbohydrate metabolism and flavonoids biosynthesis from the results of transcriptome and metabolome.

### 3.3. Ion Balance and Related Gene Expression

**Figure 4:**
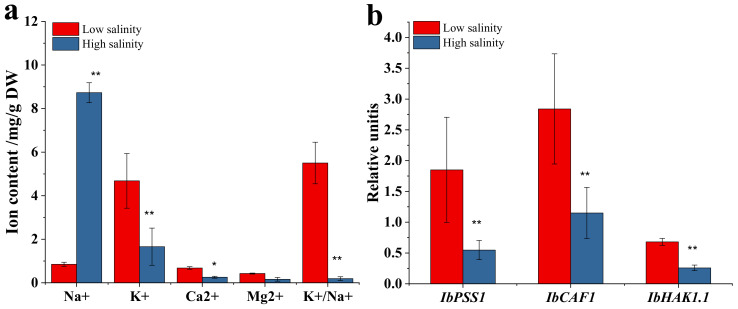
Concentrations of inorganic cations and the K^+^/Na^+^ ratio in storage root of purple sweetpotato across different treatments (**a**). Expression of genes involved in Na^+^ and K^+^ transport in storage root of purple sweetpotato across differential salinity (**b**). Columns represent mean values (±SD) of three biological replicates. Datasets with the same letter do not significantly differ (* *p <* 0.05, ** *p* < 0.01).

As we know, salinity is a bottleneck factor for ion balance in plants. Most ions including Na^+^, K^+^, Mg^2+^, and Ca^2+^ were essential to plant growth and development. However, those ions in plant cells were also susceptible to salinity, especially the Na^+^ and K^+^. In the transcriptome data (Figure 1), the result suggested that salinity might influence sodium channel activity. In further research, we evaluate the effect of salinity on the ion balance and determination of the related gene expression. Figure 4 showed the ion balance in purple sweetpotato under different salinity. As shown in the Figure 4, Na^+^ concentration in storage root increased from 0.85 mg/g in low salinity to 8.73 mg/g after high salinity applications (Figure 4a). However, K^+^ concentrations in low salinity were significantly higher than in high salinity. Obviously, the K^+^/Na^+^ for low salinity was more elevated than for high salinity. Related genes, including *IbPPS1*, *IbCAF1*, *IbHAK1.1* were determined by q-PCR. As shown result in Figure 4b, those genes were up-regulated under low salt tolerance in storage root. The *IbPSS1* could decrease cellular Na^+^ accumulation in sweetpotato [25]. In the present study, the *IbPSS1* gene level in the low salinity group was 3.6 times higher than in the high salinity group. As a common tolerance-related gene in plants, *CAF1* family gene belongs to the DEDD family, participates in the regulation of mRNA degradation, and plays a crucial role in stress response [27,28]. In this study, *IbCAF1* showed high expression in the low salinity group, about 2.19 times higher than in the high salinity group. We observed that Na^+^ transporters, such as the high-affinity Na^+^/K^+^ transporters *IbHAK1.1* were also evaluated in different salinity groups. The expression quantity in low salinity was 2.62 times higher than in high salinity. In addition, the Ca^2+^ and Mg^2+^ contents were higher than in high salinity. Those results might be suggested that purple sweetpotato could regulate genes related to the regulation of Na^+^, K^+^, and other ions concentration in cell in response to the low salinity tolerance.

### 3.4. The Effect of Low Salinity on the Oxidative Stress

**Figure 5:**
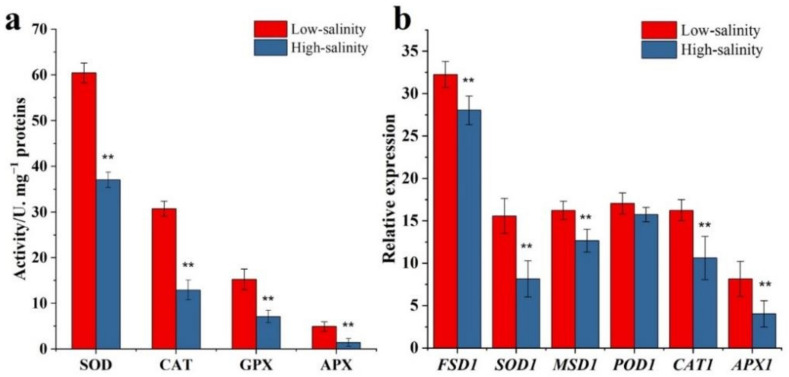
The effect of salinity on the antioxidant enzymes activity. (**a**) The activities of SDO, CAT, GPX, APX in storage root of purple sweetpotato under differential salinity. (**b**) Expression of genes of antioxidant enzyme in storage root of purple sweetpotato across differential salinity. Columns represent mean values (±SD) of three biological replicates. Datasets with the same letter do not significantly differ (** *p* < 0.01).

As we know, saltiness is induced by the cellular peroxidation of the plant. In our study, low salinity stress could keep the Na^+^/K^+^ balance, and conjecture about the low salinity could not cause cellular peroxidation. Some peroxidation indicators, including antioxidant enzyme activity and related gene expression, were evaluated in this study and are shown in Figure 5. As shown in the results in Figure 5a, the high salinity could reduce the antioxidant enzyme activities in storage roots compared to low salinity. The SOD, CAT, GPX, and APX activities in low salinity were higher by 63.15, 137.74, 114.76, and 239.04%, respectively, compared to in high salinity.

Meanwhile, we evaluated some relative gene expressions, including *FSD1*, *SOD1*, *MSD1*, *POD1*, *CAT1* and *APX1*(Figure 5b). Those genes’ expression levels decreased significantly under high salinity (*p* < 0.05). By contrast, *POD1* expression level showed no significant variation. Salinity could promote intracellular peroxidation and cause oxidation stress [29]. The result suggested that the high salinity could inhibit related genes expression level, reducing the activities of related antioxidant enzymes. Meanwhile, we found that low salinity does not promote plant oxidation and keeps superior activities of related antioxidant enzymes.

### 3.5. The Starch, Monosaccharide and Related Gene and Enzyme

**Figure 6:**
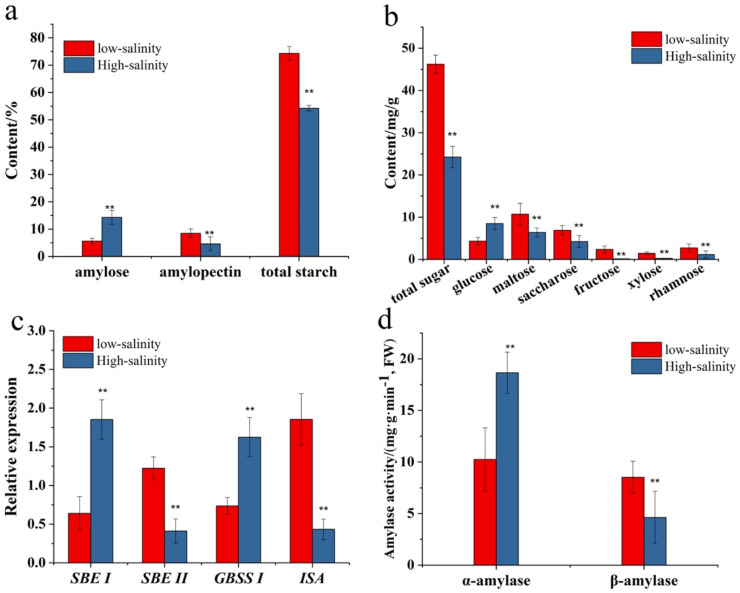
(**a**) Content of amylose, amylopectin and total starch in storage root of purple sweetpotato with different salinity. (**b**) The compose and content of total sugar and uncombined monosaccharides. (**c**) Expression of genes in starch biosynthesis pathway in storage root of purple sweetpotato across differential salinity. (**d**) Effect of salinity on the activities of α-amylase and β-amylase. Columns represent mean values (±SD) of three biological replicates. Datasets with the same letter do not significantly differ (** *p* < 0.01).

Starch and monosaccharides are the key indexes for the quality assessment of purple sweetpotato. Figure 6 showed the effect of low salinity on the starch and monosaccharide content and expression of the gene and related enzyme activity. The result shown in Figure 6a shows that the total starch in a low salinity environment was 1.44 times higher than in high salinity. For the alternative starch type, the low salinity seemed to enhance purple sweetpotato uptake of the amylopectin synthesis, and high salinity significantly promoted the amylose synthesis (*p* < 0.05). On the side of sugar, especially monosaccharides content, different salinity environments have different effects on the sugar content. As shown in Figure 6b, the total sugar in low salinity was significantly higher than in the high group at 1.77 times (Figure 6b). The glucose content in low salinity group was about 4.85 ± 0.74 mg/g, it was lower than the high salinity group (9.77 ± 1.14 mg/g). It might be related to the starch type under different salt environments. The glucose in the starch plant comes mostly from the amylolysis, and the amylose is the primary resource. The maltose content in the low salinity group was also higher than the high salinity with 1.59 times (*p <* 0.05). Meanwhile, the saccharose in low salinity group has 2.1 times higher than in the high salinity. In addition, in low salinity group, the content of fructose, xylose and rhamnose were also significantly higher than high salinity.

To verify the sugar result, the related genes, including *SBE I*, *SBE II*, *GBSS I* and *ISA* were determined by RT-qPCR (Figure 6c). As shown in the Figure 6c, the *SBE II* and *ISA* expression in the low salinity was significantly at least 2 times higher than high salinity group. Some reports showed that the *SBE II* and *ISA* were the critical genes in the amylopectin synthesis in the plant. In contrast, the *SBE I* and *GBSS I* was the key genes in the amylose synthesis. In this study, low salinity could promote the expression of *SBE I* and *GBSS I*. This result validated the effect of low salinity on the synthesis of amylopectin.

Meanwhile, those results also revealed a significantly positive correlation between low salinity and total starch content. As shown in Figure 6d, the low salinity can increase the activity of β-amylase, which is the critical enzyme for the catalyzed conversion of starch to maltose. In contrast to this, the α-amylase activity significantly increased under high salinity. The enzyme α-amylase is the key to the catalyzed conversion of starch to glucose. That result was consistent with the monosaccharide’s composition and content.

### 3.6. The Effect of Salinity on the Sugar Metabolites of Purple Sweetpotato

**Figure 7:**
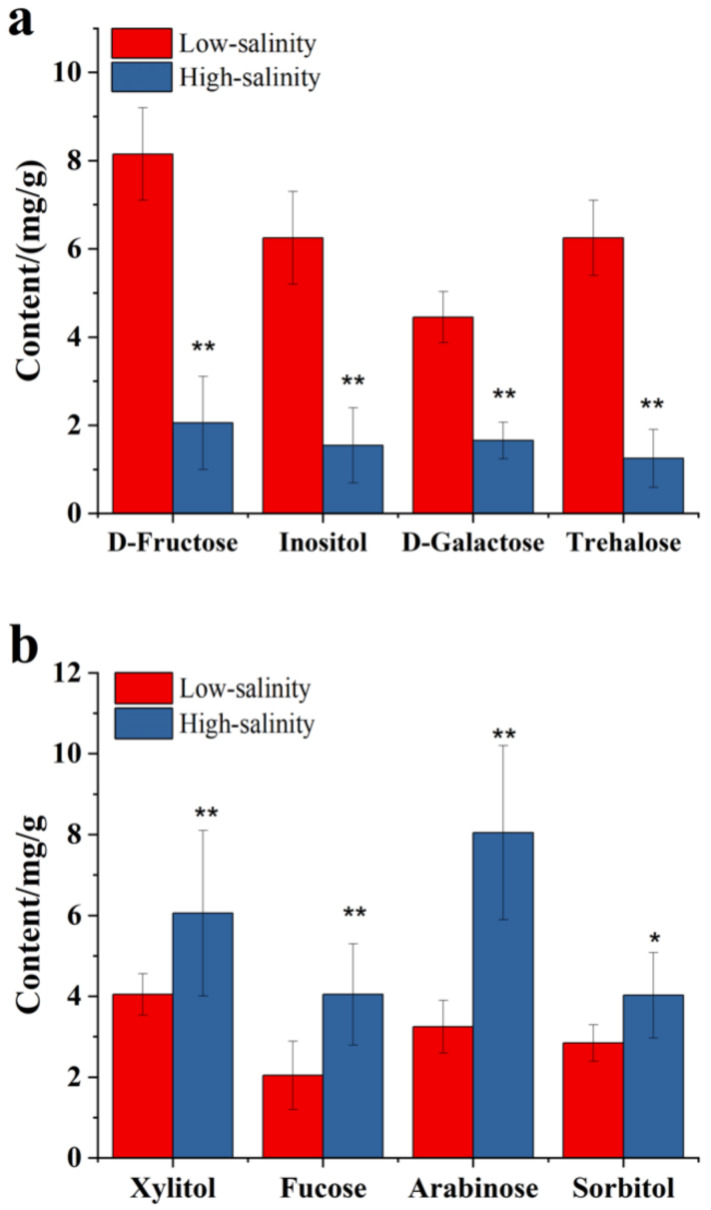
The quantitative contents of sugar metabolites in salt-treated purple sweetpotato. (**a**) The content of D-fructose, inositol, D-galactose and trehalose between low and high salinity. (**b**) The content of xylitol, fucose, arabinose and sorbitol in storage root of purple sweetpotato in low and high salinity treated. Columns represent mean values (±SD) of three biological replicates. Datasets with the same letter do not significantly differ (* *p <* 0.05, ** *p* < 0.01).

To better investigate the effect of salinity stress on the profiles of sugar metabolites in storage root of purple sweetpotato, the quantitative contents of eight different sugars except for glucose, maltose, fructose, xylose and rhamnose in Figure 7 were measured in salinity treated by GC–MS. As shown in Figure 7, the content of D-fructose, inositol, D-galactose, and trehalose were significantly increased under the low salinity. Those sugar metabolites were about 3.9, 3.4, 2.3, and 3.9 times higher than in high salinity. Meanwhile, the contents of xylitol, fucose, arabinose and sorbitol were significantly increased under high salinity. Those sugar metabolites were about 1.5, 1.9, 2.1 and 1.3 times higher, respectively, than in low salinity. The above results suggest that the sugar metabolites in purple sweetpotato could be affected by low salinity. The sugar metabolites might reflect the quality of purple sweetpotato under salinity.

### 3.7. The Effect of Salinity on the Composition and Content of Anthocyanins, Flavonoids, and Other Polyphenols

**Figure 8:**
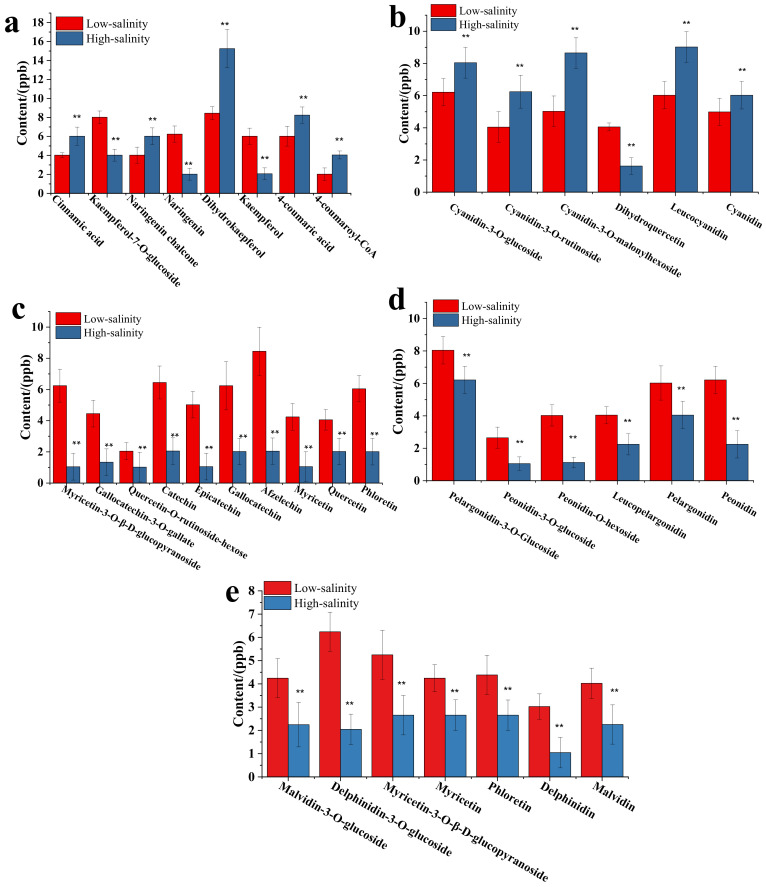
Effect of the salinity influence on polyphenols accumulation in low and high groups. (**a**) The salinity influences the accumulation of cinnamic acid, kaempferol-7-O-glucoside, naringenin chalcone, naringenin, dihydrokaempferol, kaempferol, 4-coumaric acid, and 4-coumaroyl-CoA; (**b**) The salinity influences the leucocyanidin, dihydroquercetin, cyanidin and its family ingredients. (**c**) The effect of salinity on the content of other anthocyanins. (**d**) The effect of salinity on the content of peonidin, pelargonidin, leucopelargonidin, peonidin and its family ingredients. (**e**) Effect of differential salinity on the content of Malvidin, delphinidin, phloretin, myricetin, myricetin-3-O-β-glucopyranosided and its glycoside compound. Datasets with the same letter do not significantly differ (** *p* < 0.01).

**Figure 9:**
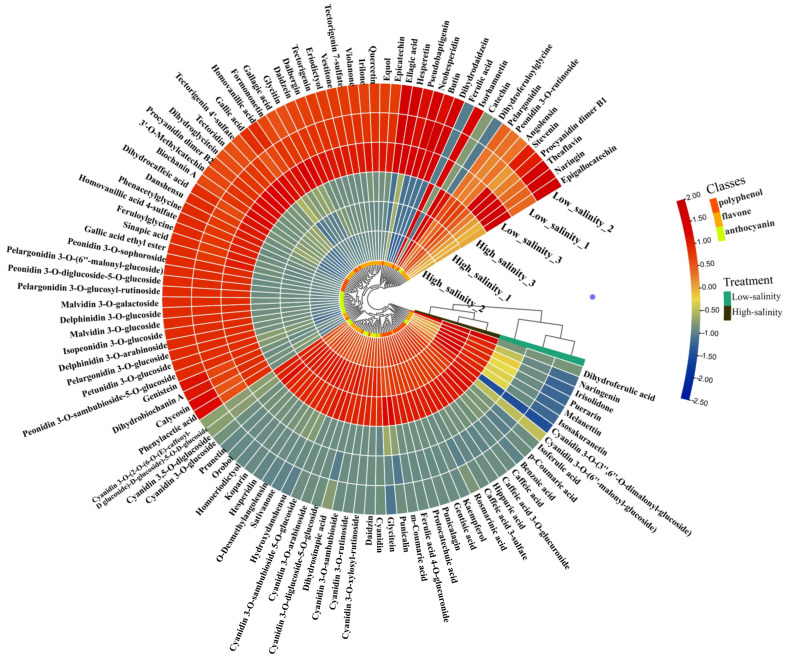
The effect of salinity on the composition and content of anthocyanins, flavonoids, and other polyphenols.

**Figure 10:**
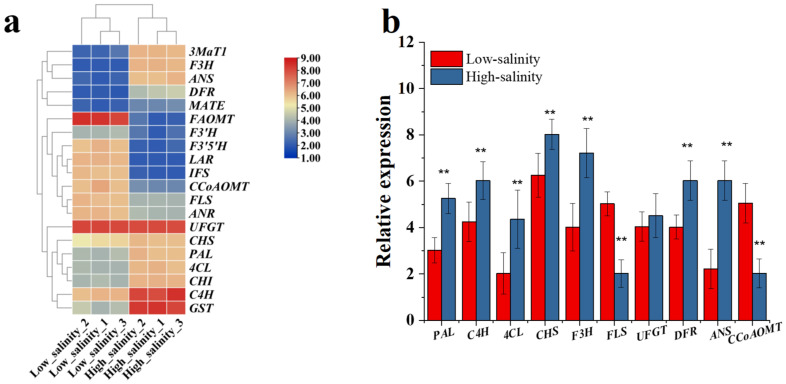
Absence of the polyphenol synthesis genes expression in different salinity groups. (**a**) Expression of major genes involved in the polyphenol synthesis in response to the different salinity. A heatmap is constructed by FPKM (Z-score method) in storage root of purple sweetpotato. (**b**) Changes of key genes validated by RT-PCR compared to RNA-Seq analysis. Datasets with the same letter do not significantly differ (** *p* < 0.01).

As we know, anthocyanin, flavonoid and polyphenol were the main chemical ingredients in the purple sweetpotato storage root. In the preliminary result (Figure 1 and Figure 2), we confirmed that the salinity influences the anthocyanidin, flavonoid and phenylethane biosynthesis. To explore the impact of salinity on the composition and content of anthocyanins, flavonoids and other polyphenols. LC–TOFMS was used in the determination of anthocyanins, flavonoids and polyphenols and the identification result showed in the Figure 8 and Figure 9. From Figure 8, it is clear that the content of most flavones and phenolic acids increased under the low salinity. However, anthocyanin, especially the cyanidin family, increased under the high salinity. To investigate the role of salinity in regulating polyphenol biosynthesis at the transcriptional level, genes involved in salinity transduction and polyphenol biosynthesis pathway were analyzed using RNA-Seq and RT-PCR (Figure 10). Major genes associated with polyphenol expression, especially *3MaT1*, *F3H*, *ANS*, *PAL*, *4CL*, *CHS*, *C4H*, DFR, and *GST* were significantly increased in high salinity. In contrast, significant increases in *FAOMT*, *CCoAOMT* and *FLS* were detected in purple sweetpotato under low salinity.

Determination of some nodal ingredients in the flavonoid and anthocyanin biosynthesis and shown in the Figure 8. As shown in Figure 8a, the cinnamic acid, naringenin chalcone, dihydrokaempferol, 4-coumaric acid and 4-coumaroyl-CoA were significantly increased and the contents of kaempferol-7-O-glucoside, naringenin, and kaempferol were significantly decreased in high salinity treatment compared to low salinity. In particular, the content of dihydrokaempferol under high salinity was increased by 86.74% compared with low salinity. Dihydrokaempferol is the critical precursor coordination compound that induces anthocyanin synthesis, including cyanidin, pelargonidin, and leucodelphinidin. The result in Figure 8b showed the effect of salinity on the content of typical ingredients in the biosynthesis of cyanidin. Significantly, it showed that the high salinity promotes the synthesis of cyanidin and its glycoside derivatives. On the contrary, low salinity positively affected the biosynthetic pathway of pelargonidin, peonidin, delphinidin, and myricetin (Figure 8c–e).

**Figure 11:**
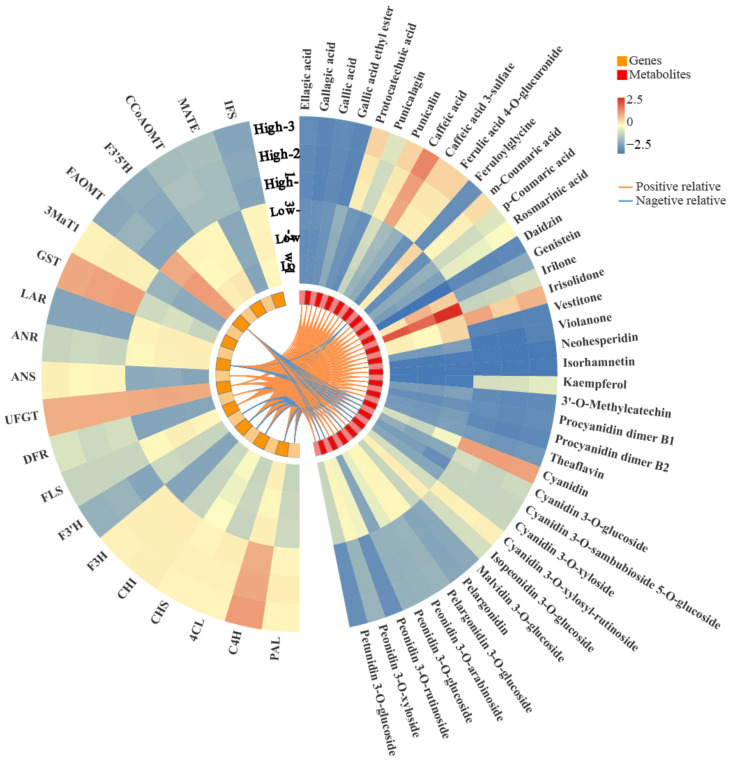
Correlation network of representative genes (**left**) and polyphenol ingredients (**right**) in response to the salinity signal.

Correlation analysis was performed for major genes in the polyphenol biosynthesis pathway and major ingredients (Figure 11). As shown in the result, the genes including *3MaT1*, *ANR*, *UFGT*, *FLS*, *F3′H*, *F3H*, *CHS*, *C4H* and *PAL* were the major genes and plays a role in mediating salinity-regulated polyphenol biosynthesis in purple sweetpotato. Meanwhile, the *ANS*, *DFR*, *FLS* involved in the polyphenol biosynthesis were positively associated with quercetin, pelargonidin, myricetin and dihydromyricetin anthocyanins.

## 4. Discussion

Eliciting plants involves the application of biological, physical and chemical factors that induce stressful conditions and physiological changes of crops. Most studies have proved that this technique is helpful in increasing crop quality and health benefits [30]. The treatments which can be classified as stressors are generally biotic and abiotic. UV-B and salinity were the common treatments in agricultural production. As we know, soil and water salinization is a global problem and an essential factor affecting crop growth [31]. Under excessive NaCl, plants can exhibit morphological, biochemical, physiological, and metabolic changes that severely affect growth [32]. As we know, ion channels play an essential role in mineral uptake, translocation and salt resistance. Na and K ions were the typical ions in this process. In this study, the Na^+^ concentration in the high salinity group increased 90.26% rather than low salinity (Figure 4a). The K^+^/Na^+^ value for low salinity was more greatly elevated than for high salinity, suggesting that purple sweetpotato could keep a preferable ability to regulate Na^+^ and K^+^ under low salinity. A further result showed that high salinity could disorient purple sweetpotatoes to lose their regulating ability, leading to ionic imbalance. *IbPSS1*, *IbCAF1* and *IbHAK1.1* were the key genes in regulating Na^+^ and K^+^. In our study, those three gene expression levels in the low salinity were higher than in the high salinity group (Figure 4b). This result further indicates the high salinity could cause an ionic imbalance. The acute imbalance of sodium ions will damage the cell and cause cell peroxidation. In particular, different antioxidant systems are involved in plant protection, including enzymatic (such as superoxide dismutase (SOD) and catalase (CAT) enzymes) and non-enzymatic reactions [33]. Some studies consider that NaCl within a certain concentration range could increase the antioxidant activity of crops. For example, Chisari reported that salinity levels up to 4.8 dSm^−1^ could reduce the activity of polyphenol oxidase (PPO) and peroxidase (POD) to protect phenolic compounds from degradation [34]. Moya found that the salinity could increase tomatoes’ antioxidant activity by increasing the bioactivity compounds content level [35]. Moreover, Ehret reported a significant effect of salinity on tomato fruit antioxidants, including lutein, lycopene, and vitamin C [36]. In this study, the antioxidant enzyme activity indicated that the low salinity could stimulate the activity of the enzyme, including SOD, CAT, GPX and APX (Figure 5a) by enhancing the related genes (such as *FSD1*, *SOD1* and so on) expression levels (Figure 5b).

In non-enzymatic reactions, polyphenol is the critical class of substances that show significantly antioxidant activity. Meanwhile, polyphenol substances are also the main components of pigments for the crop skin or fruit. Moreover, the coloration of the crop skin is an important physical property that constitutes primary consumer decision criteria. Some studies suggested that NaCl can significantly influence the pigment. For example, adding NaCl to the nutrient solution could reduce the lightness and saturation indices and increase the hue angle of a green-pigmented baby lettuce cultivar [37]. As a colored crop, consumers generally believe that the pigment reflects the nutritional value and quality of purple sweetpotato. Most pigments belong to polyphenol and are represented by anthocyanin and flavonoid compounds. Anthocyanin is recognized as the natural pigment of purple sweetpotato and belongs to flavonoids. In the present study, the low salinity significantly increased the anthocyanin biosynthesis and phenylalanine metabolism in the transcriptome and metabolome results (Figure 1, Figure 2 and Figure 3). It suggested that low salinity levels influence polyphenol biosynthesis.

Further study showed that the low salinity could increase the content levels of most phenolic acids and flavonol. Moreover, the low salinity significantly affected other anthocyanins biosynthesis except for the cyanidin family. On the contrary, the high salinity level could increase the biosynthesis of anthocyanin and its glycoside derivatives. The related gene expression results also confirmed that high salinity can promote the expression levels of biosynthetic genes of anthocyanin and its glycoside derivatives, such as *F3′H*, *DFR*, *ANS* and *UFGT* (Figure 10). Several studies have reported the beneficial effect of increased salinity on phenolic acids and flavonoids in the crop. For example, NaCl treatment could promote the total phenolic acid and flavonoid contents in cardoon leaves [38]. Falcinelli found that moderate salinity was the best compromise to increase the phenolic content of rapeseed sprouts [39]. In this study, the low salinity significantly increased the expression levels of phenolic acid biosynthetic genes, including *ANR*, *LAR*, *FLS*, *PAL*, *C4H*, *4CL* and *IFS*. The content levels of most phenolic acids, flavanols, and isoflavones, such as afzelechin, phloretin, and gallocatechin, increased under low salinity treatment (Figure 8).

In addition to secondary metabolites, the composition and content of carbohydrates are the other essential properties that affect the quality of purple sweetpotato. When subjected to abiotic stress such as drought and salt stress, plants develop mechanisms of tolerance of adaptation such as osmotic adjustment, and these mechanisms allow plants to maintain their development even under stress conditions [40]. In a sense, carbohydrates, including glucose, sucrose, and so on, are also the osmotic regulators. On the other hand, glucose and sucrose are also the flavor compounds for purple sweetpotato. The flavor of purple sweetpotato is the combined perception of taste and is related to multiple factors. Considering that, for consumers, the most critical attribute for quality evaluation is the physical appearance of a product, the flavor comes second in this process. Still, it is equally essential since it determines the recurrence of consumers’ choices and also defines whether a product will be established in the market or not. In previous studies, flavor-related compounds were considered organic acids and sugars.

However, the most critical effect of these compounds is associated with pH-regulation, which affects flavonoid biosynthesis. At the same time, sugars are used as building ingredients in phenolic acid and ascorbic acid composition [41]. In the present study, the low salinity significantly increased the total sugar and saccharose (Figure 6b). Meanwhile, other monosaccharides, including fructose, xylose, and rhamnose, increased after low salinity treatment (Figure 6b). As we know, those substances have not reflected the sweetness of the purple sweetpotato, but it might be used as osmotic regulators to respond to the salinity stress.

Meanwhile, previous results showed low salinity promotes phenolic acid and flavonoid composition and content. Considering sugar and phenolic acid as the flavor substances, types and contents could influence on the further breed selection. In present study, the low salinity increased the soluble sugar contents, especially the total sugar and the composition and content of phenolic acids and flavonoid also increased significantly. Meanwhile, the low salinity could increase the content of saccharose, which is a sweetening agent, it might indicate that the purple sweetpotato has a sweety taste under the low salinity. Meanwhile, the phenolic acid exhibits a particular flavor to a certain degree. Comprehensive consideration, the low salinity could increase the flavor substance contents and suggested that purple sweetpotato could be grown in a low salinity environment.

As we already know, salinity stress, especially high concentration of NaCl, usually inhibits plant photosynthesis and decreases crop yields. As salinity stress has negative impacts on starch synthesis and accumulation. Those negative effects result from the inhibition of photosynthesis by reducing water potential and the interference of starch development by altering many metabolic processes and activities of many synthesis enzymes [42]. Some studies reported that the salinity can induce the cellular carbon partitioning to induce the starch biosynthesis [43]. Meanwhile, some studies showed the salinity stress could reduce starch synthase and phosphorylase activity in the crop [44]. Meanwhile, some studies showed the activities of starch synthesis genes show positive correlation with the NaCl concentration [42]. In our study, the salinity significantly influenced the starch type and content. The type of starch in low salinity mainly constituted was amylopectin and amylose in high salinity. The previous results in the present study showed that salinity could change the monosaccharide composition and content. The monosaccharides, including glucose and fructose, were the base units for polysaccharides in the plant. As the most abundant polysaccharide in purple sweetpotato, starch development mainly depends on the bioproducts of photosynthesis. Salinity stress could significantly affect the synthesis and accumulation of starch in purple sweetpotato. The main reason is that salinity could change the starch units. As we know, starch is commonly composed of two types of biopolymers, amylose and amylopectin. The unit of amylose is glucose and the type of glycosidic bond mainly α-(1,4)-linkage. The amylopectin is a much larger, branched molecule containing more than half glucose units engaged in α-(1,6)-linkage [45]. It has been confirmed that those two types of starch significantly vary in their composition and functional properties. It makes those two types of starch selectively suitable for different food or non-food application and significant in various breeding.

Four main genes in the starch synthetic pathway include *SBEI*, *SBEII*, *GBSSI* and *ISA*. The GBSS enzyme was tight coupling with the starch granule to control the amylose synthesis. In the present study, the high salinity increased the *GBSSI* gene expression and amylose content levels. Meanwhile, another key enzyme in the control of starch synthesis is *SBE* and the related genes, *SBEI* and *SBEII*, separately control the synthesis of amylopectin and amylose. In this study, the *SBEI* expression level in high salinity was higher than low salinity. This corresponds to the amylopectin level being higher than low salinity and suggested that *SBEI* in purple sweetpotato was related to the amylose synthesis. Meanwhile, the *SBEII* expression level was related to the amylopectin content level and the low salinity could increase the *SBEII* expression. In addition, isoamylase (ISA), a type of starch debranching enzyme that hydrolyzes glycogen α-1.6 glycosidic bond during starch metabolism and plays a key role in amylopectin synthesis. In present study, low salinity increased the expression level of *ISA*, which is was correspondingly higher than in high salinity. Meanwhile, the α-amylase activity in low salinity was lower than high salinity and β-amylase also reveals a similar comparison. As we known, α-amylase can hydrolyze the α-1, 4-glycosidic bond to produce the maltose and hardly hydrolyze the α-1.6 glycosidic bond. Therefore, in this study, low salinity could influence the maltose content level under the control of amylase activity.

## 5. Conclusions

Although salt stress is considered an abiotic factor associated with crop productivity reduction, salinity eliciting can improve the quality of the final product, thus, a compromise is needed to find. In this study, we evaluated two different salinity concentrations on the quality of purple sweetpotato. The results showed that low salinity could influence starch synthesis, glycometabolism, and secondary metabolite metabolism to increase the flavor and quality of purple sweetpotato. High salinity could increase the anthocyanin, especially cyanidin and its family substance. The finding of this study shows the great potential of using salinity as a tool for increasing bioactive compound content in similar or higher levels compared to genetic engineering and purple sweetpotato breeding tools. Therefore, in the following study, the primary challenge for the study community will be enhancing the purple sweetpotato’s nutritional and functional attributes. Based on the above result, it could reach this target through the application of mild to moderate salinity levels, as well as through the monitoring of exposure. We should focus on identifying the genotype-environment-management proactive combination under salt stress application for a broad range of purple sweetpotatoes. Meanwhile, the molecular and physiological mechanisms to enhance of flavor and nutritional and functional attributes of purple sweetpotato also should be carried out.

## Figures and Tables

**Table 1:** Primers used for qRT-PCR and their accession numbers.

Gene Name	Primer-Forward (5′ → 3′)	Prime-Reverse (5′ → 3′)
*PSS1*	ATGACTTCCATGCTGGTGACG	CTAGCGAAAAGACAGTGTGCTTGG
*CAF1*	CCCAAGCTTATGGCTGTTGAAAAAGCTGC	GCCTCGAGTTACTAATACACTTCTAATCC
*HAK1*	ATGGTAGCCTGAACTTGCGAC	GATGGTTGGACCTCCCTTGG
*FSD1*	TTGAGCAGACAGGAGAAGCGTC	TCCCACAAGTCCAAACCGATC
*SOD1*	TCCCACAAGTCCAAACCGATC	AGCTCATGTCCTCCCTTTCCA
*MSD1*	GGTGTGGCTTGGTGTGGATAA	TGCCCAAAAGAGGAACCAAGT
*POD1*	AACTGCTGTTGAGAATGCATGC	TCCAAAATGGCCCTCCACTC
*CAT1*	CCATTTCAACTATTTTCATCGATCTTC	AAGGTCGCGTCTCGTCTCA
*APX1*	GAAGGTGCCACAAGGAACGTT	AGCCCTTCTTTTTCCCCACTC
*SBE I*	GAGCCATGACCAGGCTATTGT	ATTGGCGTCTGCACTTCTCAT
*SBE II*	ATCCAAATGCTGATGTTATGACTCG	AATGCCTGAAGGAGTGTCCATAC
*GBSS I*	GACTGCGGCATCACTGGTATTT	GAACTTAGAAATCGCAGCAT
*ISA*	TGCTCCTGAAGAAGGGCATTAC	AGTCAAACTTATCAGCGGTAGGT
*3MaT1*	TTTCCTTGGGTTCTCCTG	TTCTAACTCGGTGGTGGG
*F3H*	AAGGAAGCGTTGACCAAAGC	AAGGAAGCGTTGACCAAAGC
*ANS*	TAATGCTAGTGGGCAGCTTGAG	TAATGCTAGTGGGCAGCTTGAG
*DFR*	TGAGCATCCCAAAGCAGAAG	TGAGCATCCCAAAGCAGAAG
*MATE*	TTGTTCAGCTGGGGTACGAC	TTGTTCAGCTGGGGTACGAC
*FAMOMT*	CGAGTTTGAGGTCGATCGGT	ACAGCTTGTTTGCATCTTCCAA
*F3′H*	AAGGAAGCGTTGACCAAAGC	AAGGAAGCGTTGACCAAAGC
*F3′5′H*	TGTGGTGGTGGAAATGT	CTATAGAAAGCACCCTTCAA
*LAR*	GCAAACGTTGCAAGGGTGAATG	ACTTTGAGCAGGTTCCTCCGGGTT
*IFS*	AAACCCACGTCATGCCAGCTC	CGACAAGCACGGTCAACTTC
*CCoAMOT*	GTCTGATGGCTACTGCACCA	GTCTGATGGCTACTGCACCA
*FLS*	GCCTGTCGTTGGGTTTAGGG	GACACGGCGGGTAATAGTTGA
*ANR*	GGTGAAGCGGGTGATCTTG	TTCTCTGTCATCGTCTTGGCA
*UFGT*	AGGATTTGCAAGCGCCATTC	AGGATTTGCAAGCGCCATTC
*CHS*	AGTGCTTGTTCGAGGCTTTC	AGTGCTTGTTCGAGGCTTT
*PAL*	TGCCAGGCAATTGATCTGAG	TGCCAGGCAATTGATCTGAG
*4CL*	AAAGGATGCACGCACTTCTC	AAAGGATGCACGCACTTCTC
*CHI*	AAGTGGAACGGGAAAAGTGC	AAGTGGAACGGGAAAAGTGC
*C4H*	ATCTTGGTGAACGCTTGGTG	ATCTTGGTGAACGCTTGGTG
*GST*	TGACTCTGCTGTGGGGCTC	GGTCACAGCACCAACA

**Table 2:** The fold change of major metabolites in the storage root between low and high salinity.

Item	Class	Retention Time (min)	Metabolites	Molecular Formula	Exact Molecular Weight	Found Molecular Weight (*m*/*z*)	Error (ppm)	*p* Value	Related Content	
									Low-Salinity	High-Salinity
1	amino acid	2.74	proline	C_5_H_9_NO_2_	115.1699	115.1695	−3.47	5.22 × 10^−3^	4.06↑	8.72↑
2		6.25	glycine	C_2_H_5_NO_2_	76.0871	76.0874	3.94	2.14 × 10^−3^	6.91↑	10.05↑
3		4.55	valine	C_5_H_11_NO_2_	118.1859	118.1857	−1.69	6.24 × 10^−4^	29.8↑	40.7↑
4		4.74	alanine	C_3_H_7_NO_2_	90.12	90.1205	5.55	7.70 × 10^−5^	12.6↑	18.05↑
5		6.84	aspartic acid	C_4_H_7_NO_4_	86.1377	86.1374	−3.48	2.15 × 10^−4^	2.64↑	4.72↑
6		5.69	serine	C_3_H_7_NO_3_	106.1194	106.1192	−1.88	2.45 ×10^−3^	5.93↑	18.05↑
7		12.74	cysteine	C_3_H_7_NO_2_S	122.185	122.1853	2.46	8.40 × 10^−5^	6.91↑	11.38↑
8		14.62	phenylalanine	C_9_H_11_NO_2_	166.2538	166.2637	0.595	8.86 × 10^−3^	4.06↑	40.7↑
9		14.83	lysine	C_6_H_14_N_2_O_2_	147.2333	147.2334	0.6579	9.50 × 10^− 4^	7.08↑	8.72↑
10		15.44	tryptophan	C_11_H_12_N_2_O_2_	205.3024	205.3021	−1.46	8.54 × 10^−4^	12.6↑	15.39↑
11		18.74	cystine	C_6_H_12_N_2_O_4_S_2_	241.3462	241.346	-0.829	9.55 × 10^−3^	15.44	18.05↑
12		12.11	3-amino-isobutty acid	C_4_H_9_NO_3_	104.1529	104.1526	−2.88	9.02 × 10^−3^	8.65↓	5.14↓
13		16.55	aminomalonic acid	C_3_H_5_NO_4_	120.1029	120.1022	−5.83	6.80 × 10^−3^	9.245↓	3.11↓
14		13.83	β-alanine	C_3_H_7_NO_2_	90.1200	90.1205	5.55	8.41 × 10^−3^	4.628↓	2.74↓
15		7.62	3-hydroxypropionic acid	C_3_H_6_O_3_	91.1048	91.1047	−1.10	9.51 × 10^−4^	15.36↓	4.66↓
16		9.44	ornithine	C_5_H_12_N_2_O_2_	133.2004	133.2008	3.00	8.15 × 10^−3^	8.74↓	2.08↓
17		12.74	tyrosine	C_9_H_11_NO_3_	182.2532	182.2531	−0.549	7.56 × 10^−3^	4.99↓	1.74↓
18	Sugars	18.97	sucrose	C_12_H_22_O_11_	343.3800	343.3808	2.33	9.51 × 10^−3^	1.058↓	6.254↑
19		19.05	isomaltose	C_12_H_22_O_11_	343.3800	343.3801	0.291	5.47 × 10^−3^	6.25↓	27.11↑
20		19.77	melibiose	C_12_H_22_O_11_	343.3800	343.379	−0.291	4.53 × 10^−3^	4.81↓	12.05↓
21		18.62	fructose	C_6_H_12_O_6_	101.2046	101.2044	−1.98	6.53 × 10^−3^	5.45↓	18.64↑
22		22.76	maltose	C_12_H_22_O_11_	343.3800	343.3815	4.37	5.15 × 10^−3^	1.07↓	14.53↑
23		12.00	3, 6-Anhydro-D-galactose	C6H10O5	163.1863	163.1864	0.613	8.53 × 10^−3^	1.11↑	7.65↑
24	Organi acid	11.65	hexadecanoic acid	C_16_H_32_O_2_	257.5328	257.532	−3.11	6.54 × 10^−3^	2.74↓	2.05↑
25		15.84	linolenic acid	C_18_H_30_O_2_	279.5509	279.5503	−2.15	8.52 × 10^−3^	3.39↓	4.77↑
26		10.08	valeric acid	C_5_H_10_O_2_	103.1711	103.1715	3.88	8.45 × 10^−3^	4.05↓	6.58↑
27		17.83	pyruvic acid	C_3_H_4_O_3_	89.0889	89.0887	−2.24	6.25 × 10^−3^	2.77↓	8.14↑
28		22.68	gluconic acid	C_6_H_12_O_7_	197.2010	197.2011	0.507	4.50 × 10^−3^	3.02↓	3.59↑
29		24.05	fumaric acid	C_6_H_8_O_7_	193.1693	193.1699	3.11	3.42 × 10^−3^	2.68↓	4.87↑
30		24.77	citric acid	C_6_H_8_O_7_	193.1693	193.169	−1.55	5.14 × 10^−3^	1.44↑	4.99↑
31		20.74	malic acid	C_4_H_6_O_5_	135.1206	135.1211	3.70	6.03 × 10^−3^	2.08↑	5.64↑

## Data Availability

RNA-seq and metabolomics data will soon be submitted to the NCBI Sequence Read Archive (SRA). Additional data supporting the findings are included in the article.
